# Validity and reliability of the Brazilian Portuguese version of the Australian National University - Alzheimer’s Disease Risk Index (ANU-ADRI)

**DOI:** 10.1590/1980-57642018dn12-030003

**Published:** 2018

**Authors:** Marcus Kiiti Borges, Alessandro Ferrari Jacinto, Vanessa Albuquerque Citero

**Affiliations:** 1 MSc, Postgraduate Program in Psychiatry, Escola Paulista de Medicina, Universidade Federal de São Paulo (EPM/UNIFESP), São Paulo, SP, Brazil.; 2 PhD Associate Professor, Department of Internal Medicine, Faculdade de Medicina de Botucatu, Universidade Estadual Paulista Júlio de Mesquita Filho (UNESP), Botucatu, SP, Brazil.; 3 PhD Associate Professor, Department of Psychiatry, Escola Paulista de Medicina, Universidade Federal de São Paulo (UNIFESP), São Paulo, SP, Brazil.

**Keywords:** Alzheimer’s disease, dementia, risk factors, primary prevention, geriatric assessment, doença de Alzheimer, demência, fatores de risco, prevenção primária, avaliação geriátrica

## Abstract

The ANU-ADRI is a self-report tool that assesses risk for Alzheimer’s Disease (AD).

**Objective:**

To validate an adapted Portuguese version of this instrument and to carry out the reliability Test-Retest of the ANU-ADRI in Brazil.

**Methods:**

In this longitudinal study, the sample was formed (n=100) by two groups (A and B): each comprising 50 patients assisted by GPs (general practitioners) or specialists in dementia. All participants were cognitively healthy upon screening using the MMSE. The ANU-ADRI was applied at baseline (Test) and again within 1 week of the test (Retest).

**Results:**

There was a correlation between the mean scores of the ANU-ADRI Test and Retest (r=0.918, P<0.001). Group A had higher ANU-ADRI scores than those of group B (P<0.05). There was a moderate negative linear relation between the ANU-ADRI and MMSE scores (r= -0.353, P<0.001).

**Conclusion:**

The ANU-ADRI is a valid and reliable instrument to assess whether community-dwelling Brazilians are at greater risk for AD. Low levels of education were associated with higher risk scores on the ANU-ADRI.

## Resumo

O Índice de Risco da Doença de Alzheimer da “Australian National University” (ANU-ADRI) é uma ferramenta baseada em auto-relato que avalia o risco para Doença de Alzheimer (DA).

**Objetivo:**

Validar uma versão adaptada em português deste instrumento e realizar a confiabilidade Teste-Reteste do ANU-ADRI no Brasil.

**Métodos:**

Neste estudo longitudinal, a amostra foi composta (n=100) por 2 grupos (A e B): cada um com 50 pacientes assistidos por clínicos gerais ou especialistas em demência. Todos os participantes eram cognitivamente normais após o rastreio usando o MEEM. O ANU-ADRI foi aplicado no início (Teste) e até uma semana após o teste (Reteste).

**Resultados:**

Houve uma correlação entre a média de pontuações do ANU-ADRI no Teste e Reteste (r=0,918, P<0,001). O grupo A teve pontuações maiores no ANU-ADRI que as do grupo B (P<0,05). Houve uma relação moderada linear entre as pontuações do ANU-ADRI e MEEM (r= -0,353, P<0,001).

**Conclusão:**

ANU-ADRI é um instrumento válido e confiável para avaliar se brasileiros que vivem na comunidade estão à risco maior para DA. Baixos níveis de escolaridade foram relacionados às pontuações mais altas do ANU-ADRI.

Alzheimer’s disease (AD) is the most common form of dementia and represents a major public health problem in older people.^1^ There is a substantial body of epidemiological data regarding primary prevention of the disease.^2^ Current evidence shows multiple risk factors associated with cognitive impairment and dementia.^3^

While biological and some other factors are non-modifiable, nutritional or behavioral factors can be modified through lifestyle interventions. Early identification of modifiable risk factors and reliable estimation of the risk are important for the development of strategies to delay dementia onset.^4^

One of the challenges of interpreting findings of review studies on risk factors for AD is distinguishing factors associated with the increase or reduction of risk for AD. The most relevant non-modifiable risk factors are age and the apolipoprotein E4 (APOE4) gene variant. There is strong evidence indicating that these factors increase the risk of AD.^5^^-^ 7

Several cardiovascular (CV) and metabolic or psychiatric disorders often co-occur (e.g. hypertension, obesity, diabetes, hyperlipidemia and depression) and interact during lifetime.^8^ These disorders may be associated with an increased risk of AD. Behavioral or lifestyle-related risk factors have also been described such as low social engagement^9^^,^ 10 (loss of spouse and living alone or without a partner) or current smoking.^11^ It is therefore important to identify factors that may slow progression of AD among persons at risk and in whom the condition has already been diagnosed.

By contrast, some factors may reduce risk of dementia (e.g. involvement in cognitive activities later in life and educational attainment,^12^^-^ 14 physical activity,^10^^,^ 15 ^,^ 16 or other leisure activities^17^ that are beneficial for cognitive reserve^18^ and functioning.^19^ A healthy diet^20^ and light-to- moderate alcohol consumption^15^ have shown a possible protective role, while the Mediterranean diet^21^ may reduce the risk of dementia.

It is crucial that preventive intervention focuses on the identification of modifiable risk factors. Models have been developed for predicting dementia.^22^ Unfortunately, there is no gold standard model for dementia risk prediction from a population-based perspective.^23^ No comparable measure has been developed for risk factors measured in older adults (60 years of age or older), since CV risk factors that are assessed in a younger population were typically not the same CV risk factors used in cohorts with elderly. For example, high cholesterol and body mass index (BMI) are risk factors for AD in middle-aged patients, but both would no longer be considered risk factors in late life.^24^

In the CAIDE study, 1,449 participants were evaluated at an average age of 50 and followed up 20 years later for dementia.^25^ Based on this sample, a Mid-Life Dementia Risk Index was derived, that included age, sex, education, BMI, blood pressure, total cholesterol, physical activity, and APOE. Scores were obtained by standardizing β coefficients from a multivariable logistic model. In 2009, Barnes et al. published a Late-Life Dementia Risk Index for predicting dementia risk strata (low, moderate, or high) within a 6-year follow-up.^26^

The Cardiovascular Health Cognition Study, nested in the Cardiovascular Health Study (CHS), began in 1998-1999 and included 3608 CHS study participants, all of whom had an MRI scan and Modified Mini-Mental State Examination (3MS) scores for 1991-1994. Although the Late-Life Dementia Risk Index has somewhat stronger prediction characteristics than the Mid-Life Dementia Risk Index, its purpose was only for 6-year prediction. In addition, it required information that may not be readily obtained from individuals at the population level, including quantitative Magnetic Resonance Image (MRI) analyses. To circumvent this problem, the same investigators pooled data from four cohort studies, the CHS, the Framingham Heart Study (FHS), the Health and Retirement Study (HRS), and the Sacramento Area Latino Study on Aging (SALSA).^27^

In Australia, Anstey et al.,^28^ used a different approach to develop a risk index called the Australian National University Alzheimer’s Risk Index (ANU-ADRI) that could be inexpensively applied in populations. The ANU-ADRI has been validated in three large cohorts and this tool addresses issues involving risk and protective factors for AD.^24^

This index used the existing literature on dementia and AD risk to select risk factors that could be readily obtained by questionnaire, without clinical assessment, neuropsychological testing, genetic evaluation, or imaging studies. This approach did not rely on data from one study only, but rather estimated coefficients from existing meta-analyses of risk factors.

The use of self-reported data alone can be viewed as an advantage in terms of ease of data collection, but it can also be regarded as a liability in terms of the accuracy of exposure ascertainment. Individual scores were developed using the same technique as Kivipelto et al.,^25^ except that the standardized β coefficients were derived from odds ratios of pooled effect sizes from meta-analyses instead of from a single study. Thirty-eight potential risk factors were initially identified, 15 of which were considered to have sufficient evidence to include in the index. These variables included age (for men and women separately), education, BMI, diabetes, depressive symptoms, high cholesterol, head trauma, smoking, alcohol consumption, social engagement, physical activity, cognitive activity, fish intake, and pesticide exposure.

Satizabal et al.,^29^ found that educational levels might have contributed to the 5-year delay that they observed in the mean age at onset of clinical dementia. Participants from the Framingham Heart Study who had at least a high school diploma had showed a reduced risk of dementia but the authors concluded that “the incidence of dementia has declined over the course of three decades”. The factors contributing to this decline have not been completely identified.^29^ Chary et al.,^30^ conducted another cohort study and concluded that early predictors of dementia differ according to educational level.

We aimed to validate the ANU-ADRI for the Brazilian population by investigating whether education levels affect performance of the instrument. The Test-Retest reliability of the ANU-ADRI was determined for a sample of individuals assessed within two different settings (Primary and Secondary Care) in Brazil.

## METHODS

### Study design: longitudinal study

Participants were assessed for eligibility as follows:

- Inclusion criteria: the target sample included middle-aged individuals (40-60 years) and older adults (>60 years) living in Curitiba, Brazil.
- Exclusion criteria: participants with a history of sensory or motor deficits that would affect cognition and the assessment (e.g. hearing loss or visual impairment, parkinsonism); significant neurologic or psychiatric conditions (e.g. stroke or epilepsy, psychosis, bipolar disorder or schizophrenia); as well as other significant medical events or health problems (e.g. recent cardiovascular event, renal failure or treatment for cancer); and cognitive impairment or a diagnosis of any dementia were excluded.

Participants who met all inclusion and no exclusion criteria were invited for the baseline test between July 2015 and June 2016. All participants were recruited by GPs. Patients were referred to dementia specialists in order to exclude cases of dementia (at baseline). Briefly, the DSM-V^31^ diagnostic criteria were used for dementia.

Sample size: Data from 100 patients (n=100) were used. All participants were cognitively healthy (no evidence of cognitive decline or dementia at baseline) and divided into 2 groups by convenience (each group n=50): A= patients seen by specialists (Internal Medicine - Geriatrician and Neurologist, or Psychiatrist) - Secondary Care at the “Hospital do Idoso Zilda Arns” (HIZA) for the elderly; B= patients only seen by GPs (General Practitioners) - Primary Care at the “Ouvidor Pardinho” Community Health Center (CHC). All patients were instructed as to study goals and gave written informed consent prior to instrument evaluation.

### Assessment tools

The following instruments were used in this study:

1. Pre-test evaluation
  - Baseline questionnaire: Data base (sociodemographic questionnaire, clinical inventory): age, gender, marital status, occupation, social support, education (educational level), history of clinical disease or disability, seen by neurologist or psychiatrist, history of neurologic or psychiatric disorders, and use of psychotropic drugs.
  - Screening questionnaire: MMSE − Mini-Mental State Examination: participants were required to obtain a score >20 or >25 adjusted for level of education (cut-off scores for illiterate persons: 20; 1-4 years of education: >25; 5-8 years: >27; and 9 years or more: >28).^32^^,^ 33
2. ANU-ADRI evaluation
  - The Australian National University Alzheimer’s Disease Risk Index (ANU-ADRI):^28^ the main questionnaire was developed as an online self-report tool assessing future risk of AD. The ANU-ADRI includes 15 variables (S1 Table) : 11 risk factors and 4 protective factors, consisting of 84 items that are combined into a summary score. The ANU-ADRI is available online at *http://anuadri.anu.edu.au/for-researchers* for use by researchers.
    **S1 Table:** ANU-ADRI items and scoring (Adapted by Anstey et al.).^24^

• Age e gender (Male <65 = 0; Female <65 = 0)	• Education (>11 = 0; 8 to 11 = 3; <8 = 6)
• BMI (Normal = 0; Overweight = 2; Obese = 5)	• High cholesterol (No = 0; Yes = 3)
• Diabetes (No = 0; Yes = 3)	• TBI (No = 0; Yes = 4)
• Depression (CES-D<16 = 0; CES-D>16 = 2)	• Physical activity (Mild = 0; moderate = –2; vigorous = –3)
• Cognitive activities (Low = 0; moderate = –7; high = –6)	• Social engagement (low = 6; low to moderate = 4; moderate to high = 1; high = 0)
• Fish intake (0 to 0.25 = 0; 0.26 to 2 = –3; 2.1 to 4 = –4; ≥4.1 = –5)	• Alcohol consumption (None = 0; light to moderate = –3)
• Smoking (Current = 4; Past = 1; Never = 0)	• Pesticide Exposure (No = 0; Yes = 2)

The instrument was translated from the original language of English into Portuguese by bilingual translators, and was subsequently reviewed and evaluated as to the degree of difficulty of the translation and equivalence.^34^ All participants were assessed with the Brazilian Portuguese version of the ANU-ADRI.^35^

In the present study, test evaluation was applied via individual interviews (face-to-face) by five interviewers trained in data collection to ensure maintenance of the meaning of all items.

### Procedures

All participants provided written, informed consent before ANU-ADRI evaluation. The ANU-ADRI was applied at two time points: at baseline (Test) and within a week of the Test (Retest).

- Week 1 (ANU-ADRI Test)
- Week 2 (ANU-ADRI Retest)

Ethical considerations: the study was approved by the UNIFESP Human Research Ethics Committee (register number: 933.122) and Research Ethics Committee of the Health Department of Curitiba (register number: 1.034.372) which approved the study for feasibility of access to the research venue (at HIZA and CHC). The approved research project is available on the “Plataforma Brasil” database (CAAE registry number 38185614.51001.5505).

### Statistical analyses

All statistical analyses were performed using SPSS version 20.0 and STATA 12. The sociodemographic and clinical data were analyzed using descriptive analysis. Categorical variables were expressed as relative frequencies and absolute numbers. For continuous variables: means, quartiles, minimum, maximum and standard deviation were used. The associations between two categorical variables were tested using the Chi-square test (c2), or alternatively in cases of smaller samples,^36^ Fisher’s exact test (F) was used.

The Test-Retest reliability assessment evaluates the extent to which questionnaire scores are free from random error.^37^ The intraclass correlation coefficient (ICC) was used, where values close to 1 indicate good agreement between the answers. Based on a minimal acceptable ICC of 0.75 and on the hypothesis that the present findings would be consistent yielding an ICC of 0.90, it was established that a minimum sample size of 112 was required to attain a level of significance and power of 0.9.^38^ It was anticipated that around 10% of patients would be excluded or refuse to take part for motivational or practical reasons. Sample size analysis was performed using the PASS 2008 (Power Analysis and Sample Size System) - NCSS.

Bland-Altman^39^ analysis was used to visualize the differences and means of the two evaluations on a scatter plot. If there is no systematic bias, points around the zero value of the difference can be expected. In addition, the plot shows a confidence interval of 95% for the difference. Construct validity was used because there is no reference instrument (“gold standard” method of measurement) for comparison.^40^ Validation of the Brazilian Portuguese version of the ANU-ADRI was performed by investigating whether education influences the performance of the instrument. Higher educated older adults may have a greater cognitive reserve that can delay the clinical manifestation of dementia.^41^

In order to assess whether the ANU-ADRI is able to discriminate the individuals at risk for AD, the participants were evaluated in two different settings (Primary and Secondary Care) in Brazil. The mean scores of the two groups were compared in patients who were treated by general practitioners and by specialists in dementia. The mean scores were compared using Student’s *t*-test and Cohen’s (d) for independent samples. Effect size complements the statistical hypothesis testing and plays an important role in power analyses. Effect sizes for differences between means (d) was defined as small (0.2-0.4), medium (0.4-0.8), or large (>0.8).^42^

Divergent validation was evaluated based on the correlation between Mean Score on the ANU-ADRI Test/Retest and Mean Score on the MMSE, using Spearman’s correlation (r_s_) and Pearson’s correlation (r).

A significance level of 5% was adopted for all statistical tests.

## RESULTS

**Figure 1:**
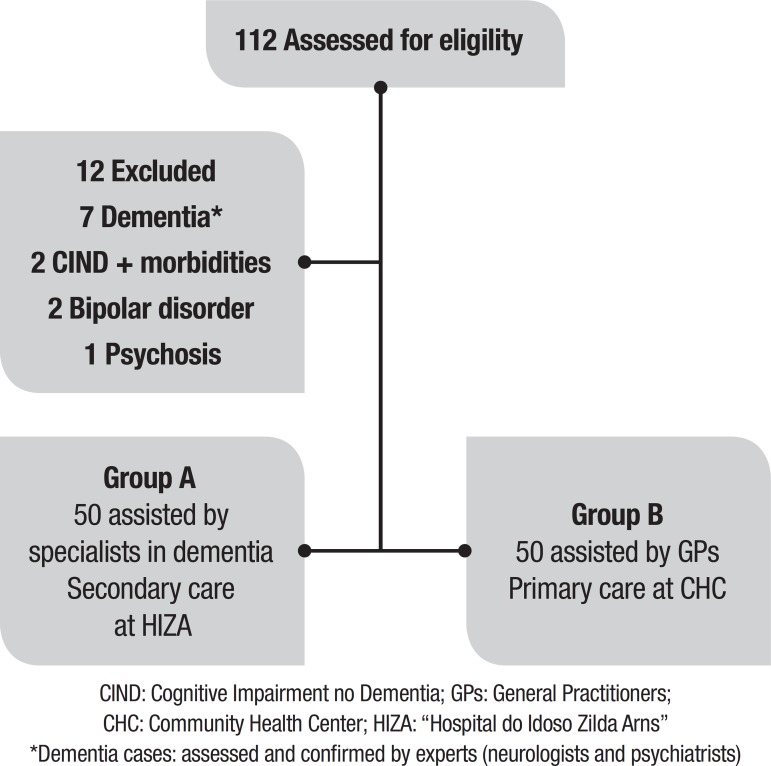
Flow diagram shows initial recruitment, exclusions and samples. CIND: Cognitive Impairment no Dementia; GPs: General Practitioners; CHC: Community Health Center; HIZA: “Hospital do Idoso Zilda Arns” ^*^Dementia cases: assessed and confirmed by experts (neurologists and psychiatrists)

A sample of 112 older adults was invited and agreed to participate in this study (Figure 1). Twelve patients were excluded: [1] two patients with cognitive impairment no dementia (CIND) and morbidities (Parkinson’s Disease or visual impairment); [2] seven patients with dementia^*^; [3] two patients with Bipolar disorder; and [4] one patient with Psychosis.

### Sociodemographic data

The mean age of the sample was 62.6 years (SD±9.8), ranging from 40 to 86. As shown in (Table 1), there was a higher proportion of women than men (67% female); 62% older adults (>60 years old); 69% with low educational level (<12 years of education); Regarding the other sociodemographic characteristics of the sample assessed: 48% married; 47% retired; 87% received social support; 68% had not received psychiatric care; and 31% had cardiovascular morbidities (only 25% without chronic medical condition).

**Table 1:** Sociodemographic characteristics of the study samples.

Variables		Groups				Total (N=100)		P-value
		A (N=50)		B (N=50)				
		N	%	N	%	N	%	
Gender	Male	20	40.0%	13	26.0%	33	33.0%	0.137^a^
	Female	30	60.0%	37	74.0%	67	67.0%	
Age	≤ 59	25	50.0%	13	26.0%	38	38.0%	0.219^b^
	60-64	9	18.0%	11	22.0%	20	20.0%	
	65-69	6	12.0%	12	24.0%	18	18.0%	
	70-74	5	10.0%	8	16.0%	13	13.0%	
	75-79	3	6.0%	4	8.0%	7	7.0%	
	80+	2	4.0%	2	4.0%	4	4.0%	
Education	No education	4	8.0%	0	0.0%	4	4.0%	0.039^b^ *
	1-4	8	16.0%	3	6.0%	11	11.0%	
	5-8	14	28.0%	10	20.0%	24	24.0%	
	9-11	13	26.0%	17	34.0%	30	30.0%	
	12+	11	22.0%	20	40.0%	31	31.0%	
Occupation	Retired	24	48.0%	23	46.0%	47	47.0%	0.054^b^
	Unemployed	6	12.0%	1	2.0%	7	7.0%	
	Qualified worker	14	28.0%	11	22.0%	25	25.0%	
	Manual worker	6	12.0%	15	30.0%	21	21.0%	
Marital status	Married	20	40.0%	28	56.0%	48	48.0%	0.239^a^
	Defacto	5	10.0%	2	4.0%	7	7.0%	
	Separated	7	14.0%	9	18.0%	16	16.0%	
	Not married	8	16.0%	7	14.0%	15	15.0%	
	Widowed	10	20.0%	4	8.0%	14	14.0%	
Social support	No	9	18.0%	4	8.0%	13	13.0%	0.137^a^
	Yes	41	82.0%	46	92.0%	87	87.0%	
Neurology assistance	No	28	56.0%	28	56.0%	56	56.0%	1.000^a^
	Yes	22	44.0%	22	44.0%	44	44.0%	
Psychiatry assistance	No	33	66.0%	35	70.0%	68	68.0%	0.668^a^
	Yes	17	34.0%	15	30.0%	32	32.0%	
Psycho (medication)	No	23	46.0%	31	62.0%	54	54.0%	0.108^a^
	Yes	27	54.0%	19	38.0%	46	46.0%	
Multimorbidities	Cardiovascular disease	19	38.0%	12	24.0%	31	31.0%	0.115^b^
	Liver disease	4	8.0%	0	0.0%	4	4.0%	
	Metabolic disease	9	18.0%	13	26.0%	22	22.0%	
	Cancer	1	2.0%	2	4.0%	3	3.0%	
	Pulmonary disease	8	16.0%	7	14.0%	15	15.0%	
	Healthy (No disease)	9	18.0%	16	32.0%	25	25.0%	

a p values calculated using Chi-Square (χ^2^) Test or

b Fisher’s exact (F) Test;

* P<0.05.

According to (Table 1), the two groups were equivalent in terms of the sociodemographic data analyzed with no statistically significant differences between them, except for the difference in distribution of education (P<0.05). The age range and the ANU-ADRI score range are given in (Tables 2 and 3), respectively. The median age was 62 years, similar to mean age. There was a statistically significant difference (P<0.05) in ANU-ADRI mean score by group (Table 3).

**Table 2:** Age range.

Mean	SD	Minimum	Maximum	Quartile 1	Median	Quartile 3	N
62.6	9.8	40.0	86.0	56.0	62.0	69.0	100

SD: standard deviation.

**Table 3:** ANU-ADRI score range.

Group	Mean	SD	Minimum	Maximum	Quartile	Median	Quartile	N	P-value^a^
A	11.0	10.8	–12.0	46.0	3.0	9.0	15.3	50	0.021*
B	6.2	9.8	–10.0	36.0	–2.0	4.5	13.0	50	

a p value calculated using Student’s t-test.

* P<0.05.

### Test-Retest reliability

The Test-Retest reliability assessment revealed good measurement reproducibility. The intraclass correlation coefficient (ICC) was 0.912 (P<0.001, 95%CI=[0.872; 0.940]), as shown in (Table 4).

**Table 4:** Intraclass correlation coefficient.

	Intraclass correlation	95% Confidence interval		F Test with true value 0	df1	df2	Sig
		Lower bound	Upper bound	Value			
Single measures	0.912*	0.872	0.940	21,721	99	100	0.00
Average measures	0.954	0.932	0.969	21,721	99	100	0.00

* P<0.001

**Figure 2:**
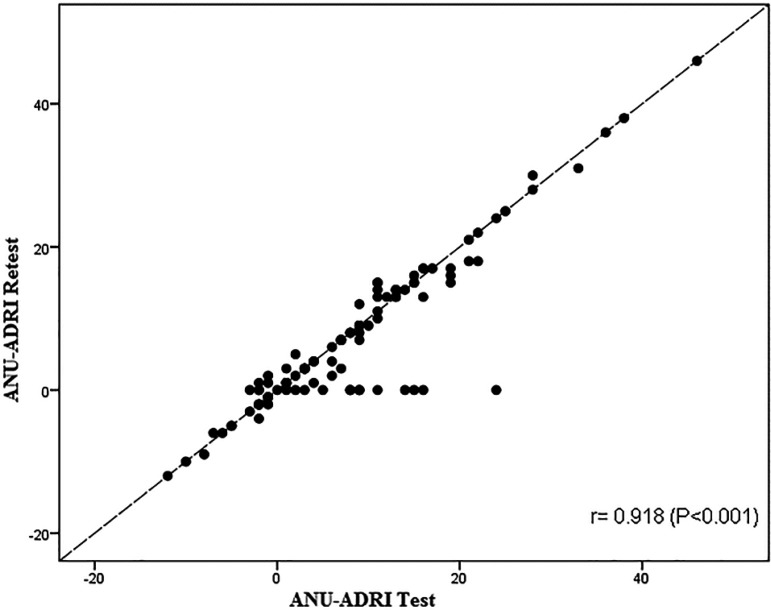
Dispersion plot of relationship between ANU-ADRI Test and Retest.

In addition, (Figure 2 and S2 Table) show a strong correlation between the mean scores on the ANU-ADRI Test and Retest (P<0.001).

**S2 Table:** The relationship between ANU-ADRI Test with Retest and MMSE.

	ANU-ADRI Test		N	ANU-ADRI Retest		N	MMSE		N
	Pearson correlation	Sig. (2-tailed)		Pearson correlation	Sig. (2-tailed)		Pearson correlation	Sig. (2-tailed)	
ANU-ADRI Test	1		100	0.918*	0.000	100	–0.353*	0.000	100
ANU-ADRI Retest	0.918*	0.000	100	1		100	–0.314*	0.001	100
MMSE	–0.353*	0.000	100	–0.314*	0.001	100	1	100	

* Correlation is significant at the 0.01 level (2-tailed).

### Bland-Altman plot

**Figure 3:**
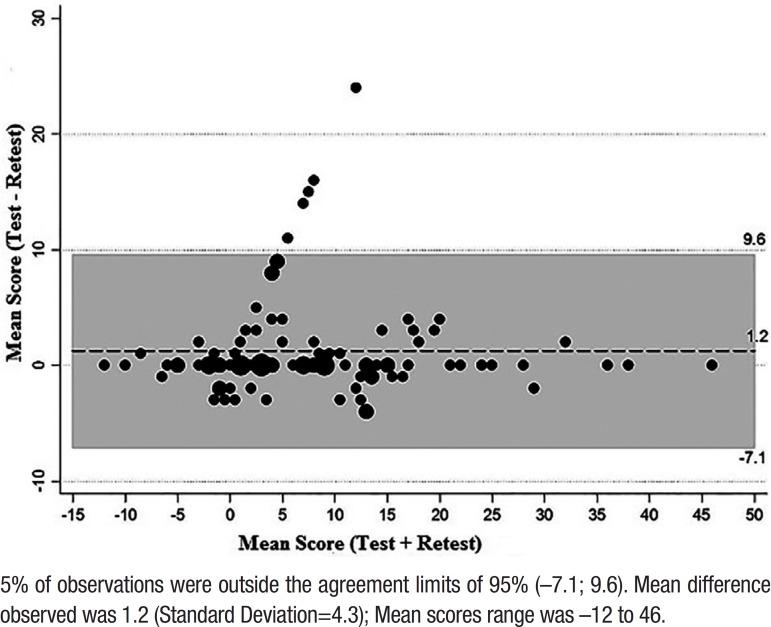
Bland-Altman plot for the two measurements of the ANU-ADRI. 5% of observations were outside the agreement limits of 95% (–7.1; 9.6). Mean difference observed was 1.2 (Standard Deviation=4.3); Mean scores range was –12 to 46.

The Bland-Altman plot shows two different measures for the same patient.^38^ Thus, the graph allows evaluation of the magnitude of discordance − by means of the difference as a function of score level (represented by the mean). The Bland-Altman plot for the two measurements of the ANU- ADRI score is shown in (Figure 3).

### Construct validity (known-groups validity)

Construct validity analyzed whether the ANU-ADRI was related to the variables it should be, thereby characterizing a valid instrument. In this approach, the ANU-ADRI questionnaire was administered to two known groups to confirm whether the hypothesized difference impact scores of the two groups.

**Figure 4:**
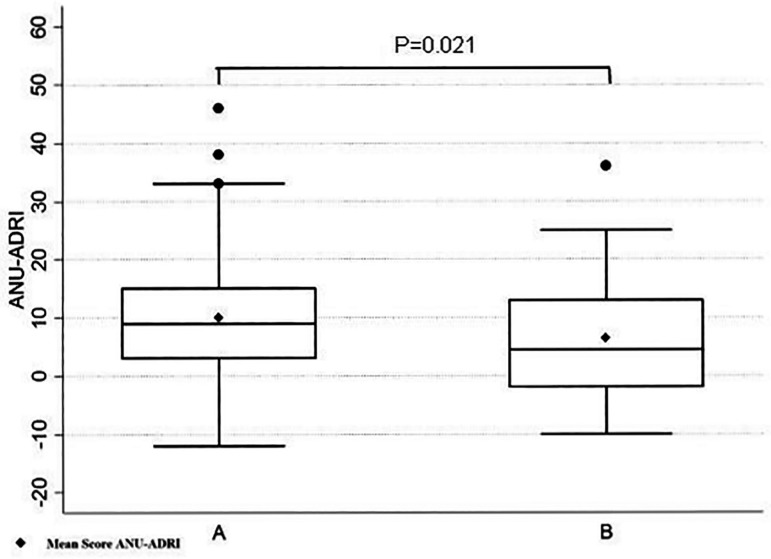
Box – Plot of the ANU-ADRI by groups (A and B).

It is notable that in group A, the percentage of individuals with 12 years or more of education was lower than for group B (P=0.039). Accordingly (Figure 4), group A had higher ANU-ADRI scores than group B (P<0.05). A higher mean score on the ANU-ADRI was found in the group of individuals with low education (<12 years of education).

Cohen’s d was used as a measure of effect size, where the comparison of two means revealed a significant difference of 0.469 (95%CI=[0.070;0.865]).

### Divergent validity

**Figure 5:**
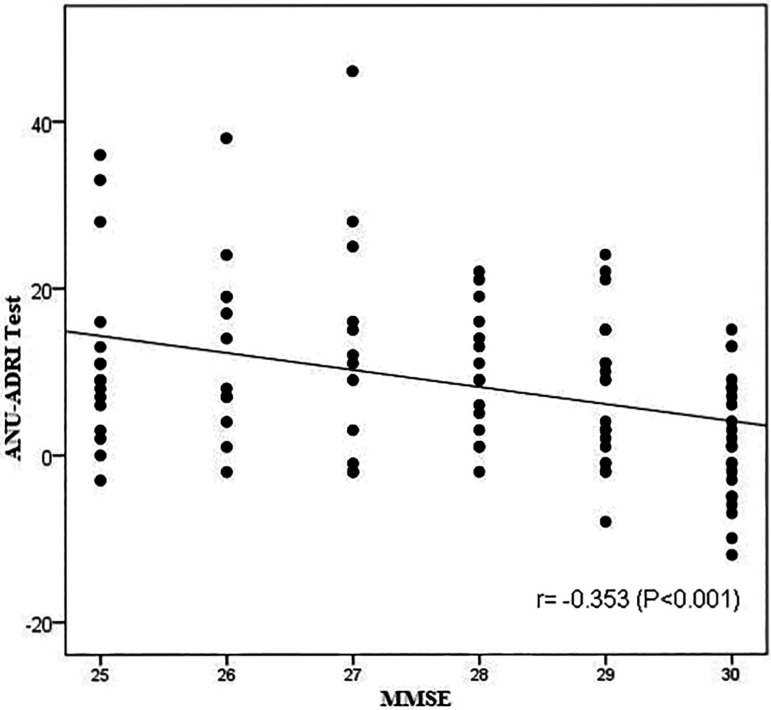
Dispersion plot of ANU-ADRI and MMSE scores.

According to (Figure 5), there was a moderate negative linear relationship between the ANU-ADRI and MMSE scores (P<0.001), indicating that the higher the MMSE score, the lower the ANU-ADRI score (less risk for developing Alzheimer’s disease). Spearman’s correlation between the two scores was r_s_= -0.350 (P<0.001).

## DISCUSSION

Strengths of this study were that the investigation included more risk factors for AD and a younger sample compared to other validation studies with samples of older adults performed in the USA and Europe. The participants were assessed by general practitioners and specialists, thus the utility of the instrument was evaluated in two settings (Primary or Secondary Care). Hence, this instrument can be used by clinicians in different settings.

The present study has several limitations. First, the samples were selected by convenience and may have been affected by selection bias in their initial recruitment. There was also the possibility that the risk scores were influenced by residual confounding. This limitation applies to other risk indices in the literature.^23^ Second, it is important to know the particularities of the sample considering the fact that respondents’ gender, age group, and level of education could have influenced the performance of the instrument. Comparing to those assessed in the other two international samples with higher educational levels (Rush Memory and Aging Study baseline age >53, and Cardiovascular Health Cognition Study >65),^24^ the present sample (two groups of individuals with lower mean age and education) had higher risk scores indicating a relatively higher risk of dementia. No comparison was made with the Kungsholmen Project^24^ (third cohort validated sample), which included participants older than 75 years and with heterogeneous level of education.

Lastly, the studies mentioned validated the ANU-ADRI in samples that were followed over time, where they were able to assess the incidence of dementia in the cohort being studied. In the present study, the authors used a construct validation, imposing significant limitations when compared to a cohort followed-up over time. The main limitation of this study is the use of only one screening tool (MMSE) to ensure cognitively normal participants.

A randomized, controlled study including a sample of middle-aged adults (mean age=55 years) with a high educational level (mean education=18 years) revealed a lower mean ANU-ADRI score (-1.38) than that found in the present samples (6.2 and 11).^43^ The author of the study suggested that the ANU-ADRI should be tested in “a target sample with lower levels of education and higher ANU-ADRI scores”.

In conclusion, the Brazilian Portuguese version of the ANU-ADRI is a reliable and valid instrument. Lower levels of education were associated with higher ANU-ADRI scores (participants at higher risk for AD) in the Brazilian population. Future research should evaluate the validity of the ANU-ADRI in large population-based samples in order to improve the effect size of validation in different contexts.
